# Structure and Function of SET and MYND Domain-Containing Proteins

**DOI:** 10.3390/ijms16011406

**Published:** 2015-01-08

**Authors:** Nicholas Spellmon, Joshua Holcomb, Laura Trescott, Nualpun Sirinupong, Zhe Yang

**Affiliations:** 1 Department of Biochemistry and Molecular Biology, Wayne State University School of Medicine, 540 East Canfield Street, Detroit, MI 48201, USA; E-Mails: nicholas.spellmon@wayne.edu (N.S.); jholcomb@med.wayne.edu (J.H.); ef6996@wayne.edu (L.T.); 2 Nutraceuticals and Functional Food Research and Development Center, Prince of Songkla University, Hat-Yai, Songkhla 90112, Thailand; E-Mail: nualpun.s@psu.ac.th

**Keywords:** SMYD (SET and MYND domain-containing proteins), structure and function, SET (Suppressor of variegation, Enhancer of Zeste, Trithorax), MYND (Myeloid-Nervy-DEAF1)

## Abstract

SET (Suppressor of variegation, Enhancer of Zeste, Trithorax) and MYND (Myeloid-Nervy-DEAF1) domain-containing proteins (SMYD) have been found to methylate a variety of histone and non-histone targets which contribute to their various roles in cell regulation including chromatin remodeling, transcription, signal transduction, and cell cycle control. During early development, SMYD proteins are believed to act as an epigenetic regulator for myogenesis and cardiomyocyte differentiation as they are abundantly expressed in cardiac and skeletal muscle. SMYD proteins are also of therapeutic interest due to the growing list of carcinomas and cardiovascular diseases linked to SMYD overexpression or dysfunction making them a putative target for drug intervention. This review will examine the biological relevance and gather all of the current structural data of SMYD proteins.

## 1. Introduction

**Figure 1:**
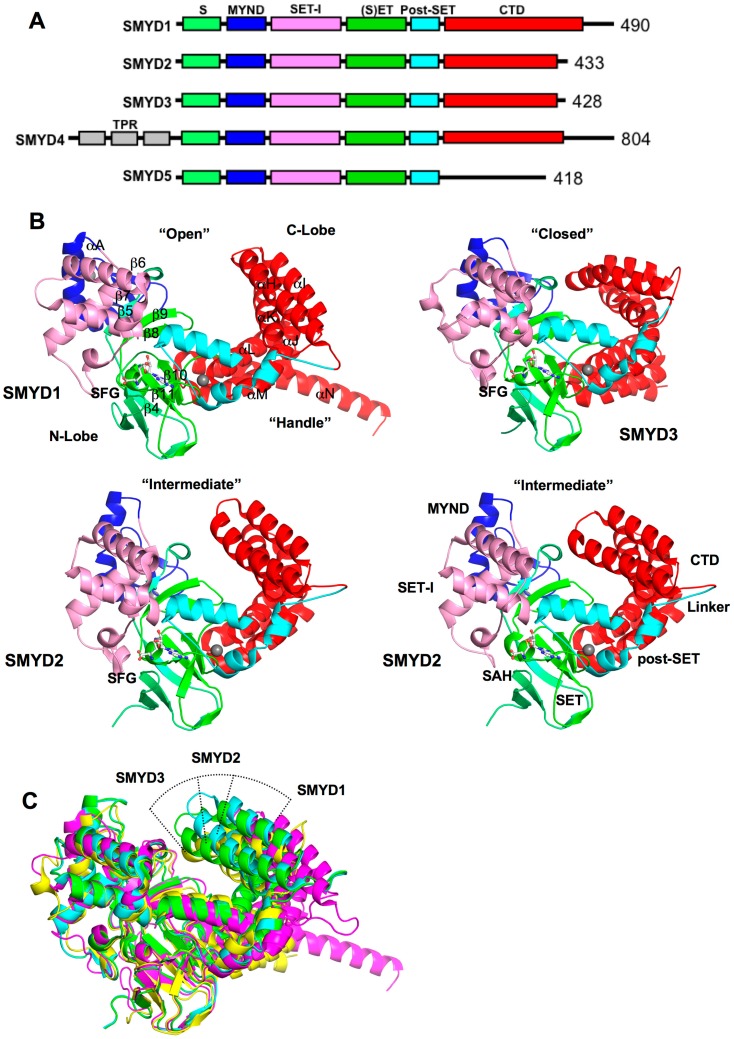
Overall structure of SET and MYND domain-containing proteins (SMYD) proteins. (**A**) Domain diagram of SMYD protein family. S, S-sequence; MYND (Myeloid, Nervy, DEAF1); SET-I, insertion SET (Su(Var)3-9, Enhancer-of-zeste, Trithorax) domain; (S)ET, core SET domain; Post-SET, SET *C*-terminal flanking domain; CTD, *C*-terminal domain; (**B**) Ribbon diagram of SMYD1 (PDB code: 3N71), SMYD2 (PDB code: 3QWV and 3QWW), and SMYD3 (PDB code: 3PDN). The S-sequence, MYND, SET-I, core SET, post-SET, and CTD are depicted in light green, blue, pink, green, cyan, and red. Secondary structures, α-helices and β-strands are labeled and numbered according to their position in the sequence. Cofactors, sinefungin (SFG) and *S*-adenosyl-l-homocysteine (SAH), are depicted in balls and sticks; and (**C**) Structural superposition of SMYD proteins: SMYD1 (magenta), SMYD2 (SFG, cyan; SAH, green), and SMYD3 (yellow). The superposition is based on the *N*-terminal lobe.

SET and MYND domain-containing proteins (SMYD) are a special class of protein lysine methyltransferases involved in methylation of histones and non-histone targets [1,2,3,4,5]. To date, there are five members from the SMYD family, SMYD1–5 (Figure 1A) [6]. Each member contains a conserved SET (Suppressor of variegation, Enhancer of Zeste, Trithorax) domain that is “split” by a Myeloid-Nervy-DEAF1 (MYND) domain [7]. The SET domain is a conserved catalytic unit for lysine methylation found in nearly all histone methyltransferases (HMT) [8]. The MYND domain is a zinc finger motif that primarily functions as a protein–protein interaction module [9,10]. Another feature is the *C*-terminal domain (CTD) found in SMYD1–4 but absent in SMYD5. Despite the lack of sequence similarity, this domain is structurally similar to tetratricopeptide repeats (TPR), which is a motif important for the binding of cochaperones with heat shock protein-90 (Hsp90) [7,11,12,13].

SMYD proteins may regulate chromatin remodeling and gene accessibility by methylating histone targets and interacting with transcription mediators. SMYD1–3 methylate H3K4, which is a methylation site promoting active transcription [1,2,7,14,15]. However, SMYD does not have an effect on global H3K4 methylation but appears to impact selective promoter regions [16,17]. SMYD1 binds directly to class I and class II histone deacetylases (HDAC) and represses transcription from an SV40-luciferase reporter [1]. SMYD2 was also found to dimethylate H3K36 *in vitro* and repress transcription through interaction with the Sin3A histone deacetylase complex [3]. However, it remains to be determined whether *in vivo* recruitment of Sin3A requires both H3K36 methylation and the presence of SMYD2. SMYD3 plays an important role in transcriptional regulation as a member of an RNA polymerase complex [2]. SMYD3 interacts with RNA polymerase II and RNA helicase HELZ suggesting that it might regulate target gene expression by facilitating transcriptional elongation. In HEK293 cells, overexpression of SMYD3 was found to up-regulate a number of genes corresponding to oncogenes, homeobox genes, and genes of the cell cycle [2]. These genes are highly expressed in colorectal and hepatocellular carcinomas [18,19]. SMYD4 was identified as a potential tumor suppressor involved in breast cancer [20]. Expression of SMYD4 partially inhibits the expression of PDGFα and the lack of SMYD4 promotes PDGFα production [20]. The *Drosophila melanogaster* homolog of SMYD4 was found to recruit the HDAC co-repressor complex and thereby aid in fly development [21]. Eri is another component of the HDAC co-repressor complex, which interacts with SMYD4 [21]. SMYD5 is known to associate with the NCoR co-repressor complex and regulate pro-inflammation genes through trimethylation of H4K20 [22]. In macrophages, the SMYD5–NCoR co-repressor complex was found to repress the expression of toll-like receptor 4 (TLR4) genes [22].

SMYD proteins methylate several non-histone targets. In the cell cycle, SMYD2 methylates p53 and retinoblastoma tumor suppressor (RB) [4,23,24]. p53 methylation by SMYD2 reduces the transactivation activity of p53 [4]. In esophageal squamous cell carcinoma (ESCC), p53 methylation and inactivation were associated with aberrant oncogenic expression of SMYD2 [25]. Additionally, SMYD2 has an anti-apoptotic effect when it methylates p53 in cardioblasts [26]. RB methylation at Lys860 is regulated during cell cycle progression and cellular differentiation [23,24]. It has been shown that RB methylation binds to the transcriptional repressor L3MBTL1 causing repression of E2F target genes [23]. In response to DNA damage, SMYD2 was also found to methylate PARP1 at lysine 528, and this methylation regulates the PARP1’s poly(ADP-ribosyl)ation activity in HeLa cells [27]. In intracellular signaling, SMYD3 targets two important kinases for methylation: MAP3K2 and vascular endothelial growth factor receptor-1 (VEGFR1). Methylation of MAP3K2 prevents PP2A phosphatase, a key negative regulator of the MAP kinase pathway, from binding to MAP3K2 [5]. Methylated MAP3K2 links SMYD3 to Ras-driven cancer promoting cell proliferation and tumorigenesis [5]. VEGFR1 methylation by SMYD3 augments VEGRF1 kinase activity, which is thought to enhance carcinogenesis [28]. Since SMYD3 is primarily found in the cytoplasm during G_0_–G_1_ arrest, it is thought that SMYD3 enhances VEGFR1 signaling when cells are at the resting state [28].

Current data have shown that SMYD proteins methylate a variety of histone and non-histone targets which contribute to their various roles in cell regulation including chromatin remodeling, transcription, signal transduction, and cell cycle control. In order to better understand how SMYD proteins interact with such an extensive yet specific range of targets, structural examination of the SMYD family has provided significant insight to the diversity of SMYD binding and function. This review will provide a thorough description of SMYD structure and function and serve to inform rational drug design process targeting this cancer-related protein family.

## 2. SMYD Structure and Function

### 2.1. Overall SMYD Structure

Crystal structures of SMYD1, SMYD2, and SMYD3 with cofactors are currently available [7,11,12,29,30,31]. Additionally, SMYD2 structures were solved with the estrogen receptor α (ERα) and p53 peptides enabling us to investigate the different interactions made between the two different substrates [29,32,33]. In all of the available SMYD structures, SMYD proteins share a homologous bilobal structure separated by a non-conserved primary sequence of variable length (Figure 1B). The *N*-terminal lobe is divided into four domains: SET, MYND, SET-I, and post-SET. The catalytic SET domain is located in the middle of the *N*-terminal lobe in proximity to the *C*-terminal lobe. The *C*-terminal lobe is organized into helices that were found to be orientated in open or closed conformations [7,11,12]. SMYD1 has the most open structure and SMYD3 has the most closed one. SMYD2 is a conformational intermediate between SMYD1 and SMYD3 (Figure 1C). The difference in the relative positions of the *N*- and *C*-terminal lobes creates different shapes for substrate binding. The structure of SMYD1 resembles an open-ended “wrench” with two lobes separated by gap. Unlike SMYD1, SMYD2 and SMYD3 form a clamshell like structure due to the absence of the *C*-terminal protruding helix.

Currently, there are no structural data for SMYD4 and SMYD5. While SMYD1–3 share well aligned domains, SMYD4 and SMYD5 are vastly different in their primary sequence (Figure 1A). SMYD4 contains an additional TPR domain before the *N*-terminal lobe, and the CTD is far extended. SMYD5 completely lacks the CTD, yet the molecular size of SMYD5 is close to SMYD2 and SMYD3. Functional implications of the differences in SMYD4 and SMYD5 are unknown, but structural data of SMYD4 and SMYD5 are of interest to address these questions.

### 2.2. SET, the Evolutionary Conserved Methyltransferase Domain

The SET domain is split by the MYND domain into two sections: the S-sequence and the core SET domain (Figure 1A). The S-sequence is a small region that may aid in cofactor binding or protein–protein interaction along with its adjacent domain, MYND [7,34]. The topology of the catalytic SET domain is well conserved between SMYD1–3, which is essentially similar to other traditional SET proteins despite the split in the primary sequence by the MYND domain [7]. SET domain often co-exists with post-SET, SET-I, and pre-SET, and together they contribute to cofactor binding, substrate binding, or the structural stability of the protein [35,36,37]. In SMYD proteins, the post-SET domain is made up of three α-helices bundled around a zinc atom coordinated by four cysteine residues. The SET-I domain along with MYND is an insertion region between the SET domain strands β5 and β8. Compared to other SET proteins like SET7 and Dim-5, this insertion region is 6–10 times larger in size in SMYD proteins [7]. The pre-SET domain is often found in other SET containing proteins, but the pre-SET region is absent in SMYD1–3 proteins. Normally, this pre-SET region packs against an equivalent β-sheet made up of β4, β10, and β11, but in SMYD proteins, this β-sheet interacts with residues from the CTD in the αM–αN loop (Figure 1B). Interestingly, SMYD4 contains TPR repeats flanking the *N*-terminus of the *N*-terminal lobe (Figure 1A). This new region may introduce a pre-SET domain or add a third lobe to the overall structure.

### 2.3. MYND, the Zinc Finger Motif

**Figure 2:**
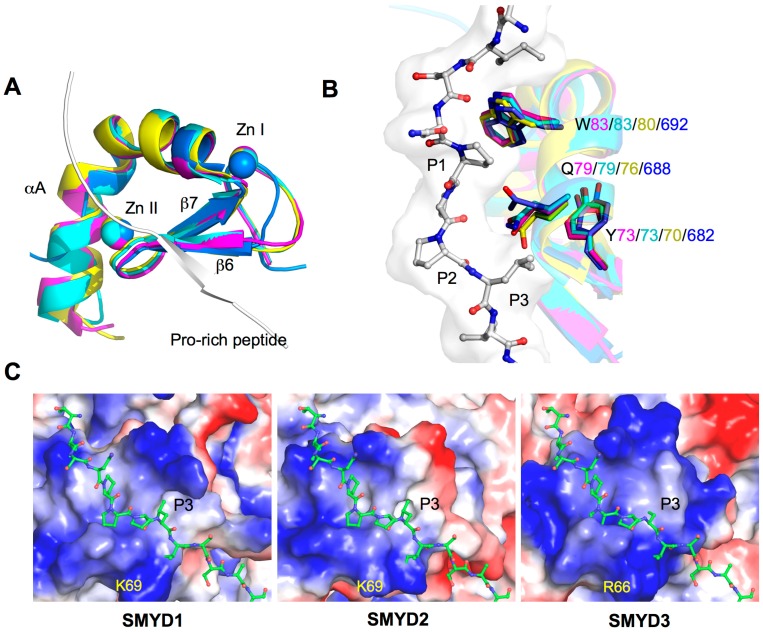
Structure of MYND domains. (**A**) Structural superposition of the MYND domains of SMYD and AML1/ETO (PDB code: 2ODD). MYND is represented by ribbon and colored in magenta (SMYD1), cyan (SMYD2), yellow (SMYD3), and blue (AML1/ETO). Proline-rich peptide bound to AML1/ETO is depicted by ribbon; (**B**) Superposition of the peptide binding pockets. Putative peptide interacting residues are colored according to the scheme in (**A**). The proline-rich peptide bound to AML1/ETO is depicted by balls-and-sticks; and (**C**) Surface representation of the MYND domains. Coloring is according to the electrostatic potential: red, white, and blue correspond to negative, neutral, and positive potential, respectively. The vacuum electrostatics/protein contact potential was generated by PyMOL. The proline-rich peptide, represented by balls-and-sticks, is modeled by superposition of the MYND domain of SMYD and AML1/ETO.

MYND domain is a zinc finger motif identified to bind to proline-rich regions serving as a protein–protein interaction module [9,10,38]. In SMYD proteins, the MYND domain is part of the *N*-terminal lobe that interacts with the catalytic SET domain, but it does not participate in substrate or cofactor binding (Figure 1B) [7,11,12]. Consistently, deletion of the MYND domain does not affect the methyltransferase activity of SMYD2 *in vitro*, suggesting that the MYND domain is dispensable in methylation [14]. Despite the high sequence similarity to LIM (Lin11-Isl1-Mec3) domains, the MYND domain exhibits a different type of fold. The secondary structure of the MYND domain adopts a β–β–α topology, which is structurally similar to some PHD (Plant Homeo Domain) and RING motifs (Figure 2A). Although the MYND domains from AML1/ETO and SMYD proteins are only 30% identical in the primary sequence, the backbone and chelating zinc centers of the MYND are well superimposed (Figure 2A). The two structures share two anti-parallel β-strands (β6 and β7) and a small kinked α-helix (αA) that organize around two zinc atoms. Seven cysteine residues and one histidine are centered around the two zinc ions in a C4C2HC arrangement.

The MYND domain from AML1/ETO is known to bind to a PPPLI motif, and in SMYD1 and SMYD2, the MYND domain can interact with proteins with a similar proline-rich sequence [9,34,39]. SMYD1 binds to the muscle-specific transcription factor skNAC via a PPLIP motif [34]. In a previous yeast two-hybrid study, SMYD2 was found to interact with five different proteins possessing a PXLXP motif [39]. To date, a MYND-binding partner for SMYD3 has not been identified, but the MYND structural similarities certainly suggest a proline-rich peptide-binding site for SMYD3. Three conserved and highly superimposed residues in SMYD3 (Trp80, Gln76, Tyr70) and in SMYD1–2 (Trp83, Gln79, Tyr73) may contribute to the binding of the proline-rich peptide (PXLXP) (Figure 2B). The tryptophan residue may pack against the first proline (P1), and the remaining glutamine and tyrosine residues may form a hydrophobic pocket for leucine (P3) to bind. The S-sequence in SMYD1 is also involved in binding to the skNAC proline-rich peptide [34], suggesting that regions other than the MYND domain may also play a role in determining binding specificity.

Electrostatic surface analysis shows the MYND domain is highly positively charged in SMYD1–3 (Figure 2C). This positively charged surface likely contributes to a protein–DNA interaction. SMYD binding to DNA was first identified in SMYD3, and binding to a specific DNA motif, 5'-CCCTCC-3', was found to regulate transcription of SMYD3 target genes such as *Nkx2.8* [2]. Mutation of Arg66 within the MYND domain disrupted DNA binding of SMYD3 and abolished a DNA-induced increase in SMYD3 methyltransferase activity [40]. Interestingly, Arg66 appears to superimpose with similar conserved Lys69 in both SMYD1 and SMYD2, and the positively charged surface across the MYND domain is well observed across SMYD1–3 (Figure 2C). This certainly suggests that SMYD1 and SMYD2 are also involved in DNA binding. A recent study shows that SMYD2 binds to the promoter region of TACC2 and regulates TACC2 expression at a site different from the binding site for SMYD3 [14].

The exact nature of DNA binding in SMYD proteins is unknown, and how DNA binding affects the activity of SMYD proteins is yet to be identified. Structural studies of SMYD–DNA complexes are of interest to address these questions. Additionally, the overlap of the positively charged surface and proline-rich peptide-binding site in the MYND domain (Figure 2C) raises intriguing questions regarding whether the peptide and DNA binding are mutually exclusive and what are the functional roles of such a scenario in the context of transcriptional regulation. In many cases, binding of SMYD3 to the promoter region of its target genes is associated with both H3K4 trimethylation and gene activation [17,41,42,43]. Surprisingly, SMYD3 shows virtually no activity towards H3K4 *in vitro* compared to other targets such as H4K5 or MAP3K2 [5,44]. This inconsistency suggests that DNA binding may induce a conformational change in SMYD3 that may subsequently affect substrate binding and specificity. Such a model remains to be determined, but the ability of SMYD2 to undergo a conformational change that alters the shape of the substrate-binding site provides a rationalization for this possibility [12].

### 2.4. Cofactor Binding Pocket

**Figure 3:**
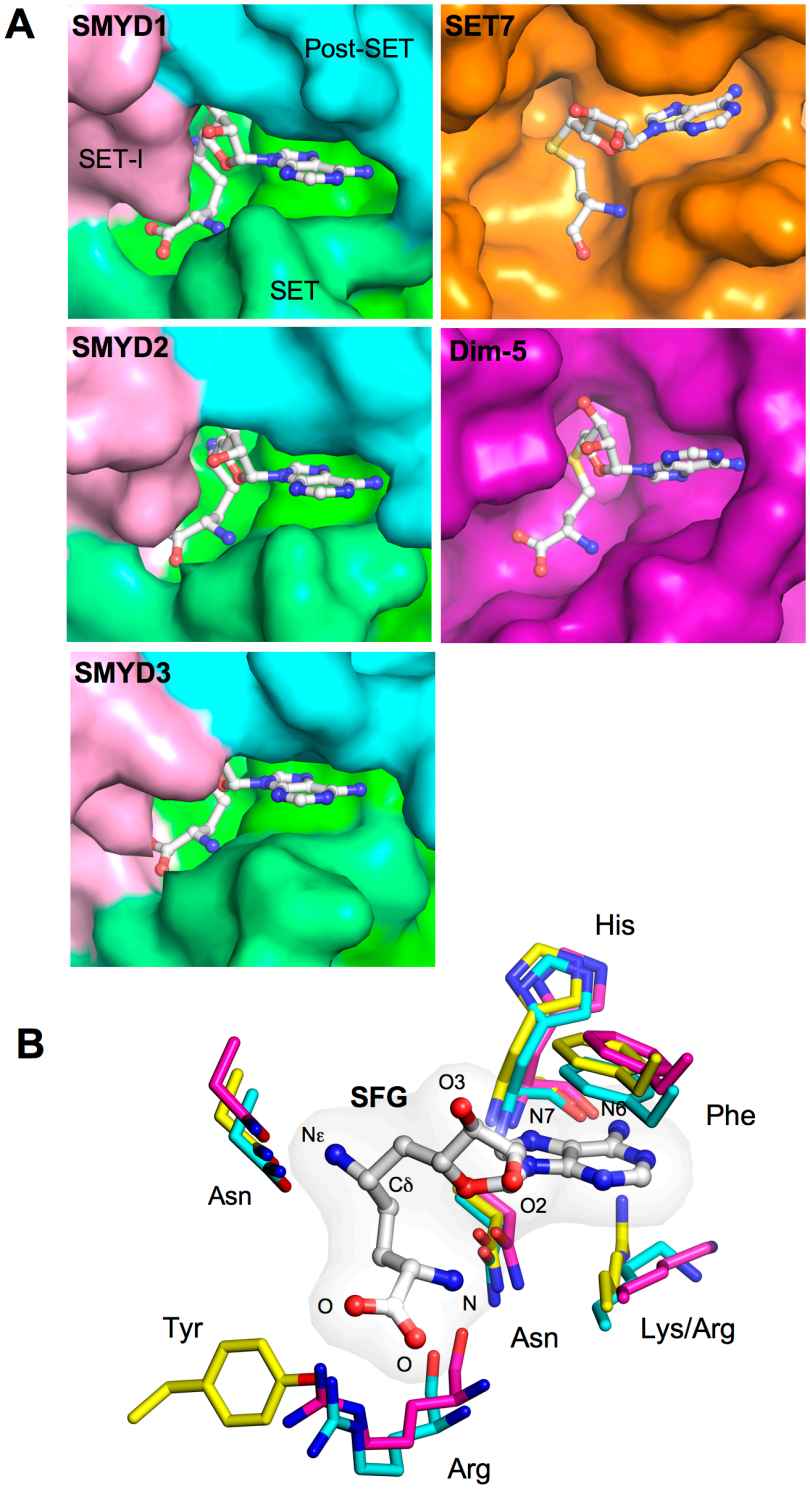
Cofactor binding pocket. (**A**) Surface representation of the cofactor-binding pocket of SMYD1–3, SET7 (PDB code: 1O9S), and Dim-5 (PDB code: 1PEG). The surface of SMYD proteins is colored according to domains. Bound SAH or SFG is depicted by sticks with the carbon atoms colored in white; (**B**) Superposition of the cofactor binding sites. SMYD residues are represented by sticks with the carbon atoms colored according to the scheme in Figure 1C. Cofactor is depicted by balls-and-sticks overlaid with translucent molecular surface.

The SET-I, SET, and post-SET domains create a deep surface pocket allowing the L-shaped cofactors, *S*-adenosyl-l-homocysteine (SAH) and sinefungin (SFG) to bind (Figure 3A). Several cofactor and pocket interactions are shared among the SMYD family (Figure 3B). The adenine moiety of SAH or SFG is sandwiched between a conserved benzyl phenylalanine and aliphatic lysine or arginine side chain. The purine atoms N6 and N7 form a hydrogen bond to the carboxyl and amide groups of a conserved histidine residue, but the ribose hydroxyl groups form hydrogen bonds with somewhat similar neighboring residues among the SMYD family. At the positively charged amino group, a similar triangular array of hydrogen bonds is formed with the carbonyl oxygens from arginine and lysine (asparagine in SMYD3) and the amide Oδ from a separate asparagine. In the middle of cofactors, two backbone carbonyls and the side chain oxygens from conserved tyrosine and asparagine surround the Sδ atom in SAH or the C–NH_2_ amine group in sinefungin. The C–NH_2_ amine group of sinefungin corresponds to the S–CH_3_ sulfonium group in *S*-adenosyl-l-methionine (SAM). Some of the aforementioned surrounding oxygens are responsible for hydrogen bonding with the C–NH_2_ amine group in sinefungin, and the same interaction is thought to contribute to destabilizing the active methyl group during enzymatic methylation [7,33].

Large differences were observed at the carboxylate moiety of cofactors. In SMYD1–2, the carboxylate moiety is stabilized by a salt-bridge interaction with an arginine guanido group, but in SMYD3, this electrostatic interaction is substituted by a hydrogen bond to a tyrosine residue from a non-equivalent location (Figure 3B). This hydrogen bonding in SMYD3 represents an unusual variation, as the replaced electrostatic interaction is present in most SET containing proteins [7,12,45,46]. As a result, the cofactor binding sites of SMYD1 and SMYD2 are more similar to one another than they are to the SMYD3 structure. However, all SMYD proteins have a nearly buried cofactor-binding site compared to SET7 and Dim-5. The bound cofactors share similar interactions in these proteins, but the large SET-I domain of SMYD proteins creates a nearly buried cofactor conformation (Figure 3A). This buried cofactor conformation, however, does not affect the enzymatic activity of SMYD1. Mutation of the SET-I residues responsible for the buried conformation only had modest effects on H3K4 methylation activity [7]. However, mutation of the S-sequence residues responsible for the adenine moiety binding completely abolished the enzymatic activity [7]. This suggests that the split S-sequence is an integral part of the SET domain contributing to cofactor binding.

### 2.5. Substrate Peptide Binding Site

**Figure 4:**
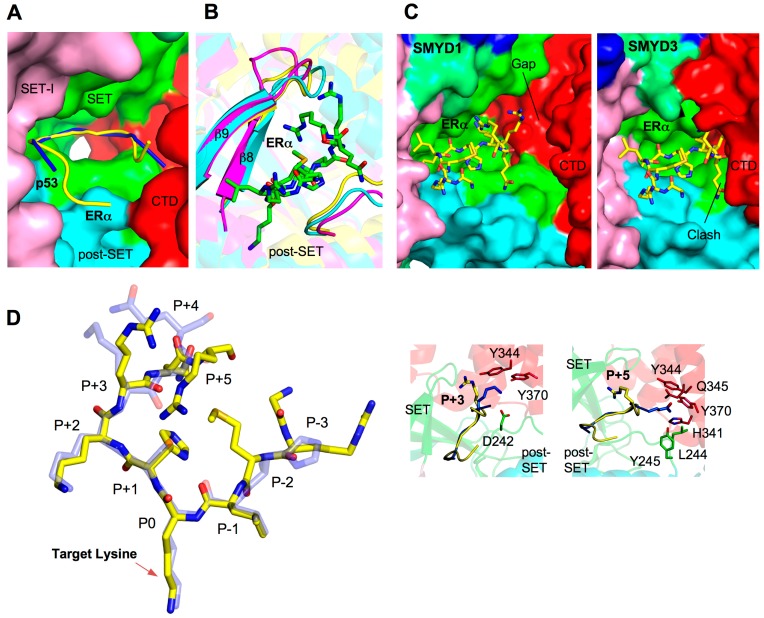
Substrate binding site. (**A**) Surface representation of SMYD2 substrate binding site. The surface is colored according to domains. ERα and p53 peptides are depicted by ribbons and colored in yellow and blue respectively; (**B**) Superposition of the substrate binding clefts. SMYD residues are represented by ribbons and colored according to the scheme in Figure 1C. ERα peptide is shown in balls-and-sticks colored in green; (**C**) Surface representation of the substrate-binding site of SMYD1 and SMYD3. The ERα peptide, represented by sticks, is modeled by superposition with the *N*-terminal lobe of SMYD2; and (**D**) Superposition of the SMYD2-bound ERα (yellow; PDB code: 4O6F) and p53 peptides (light blue; PDB code: 3TG5). Position 0 refers to the target lysine. Detailed structural and binding differences at position +3 and +5 are shown in callout boxes. Peptide-interacting SMYD2 residues are colored according to domains.

To date, there are two SMYD–substrate complex structures available, SMYD2–ERα and SMYD2–p53 [29,32,33]. These structures have provided significant insight into the substrate binding and broad substrate specificity of the SMYD family. The ERα and p53 peptides bind to SMYD2 in a U-shape conformation (Figure 4A). The peptides are clamped between the *N*- and *C*-terminal lobes with the target lysine inserted into the lysine access channel. Residues contributing to peptide binding mainly come from the β8–β9 hairpin and a loop preceding the post-SET domain. The structure and residues at the turn of the β-hairpin vary significantly among the SMYD family, but in SMYD2 this region is important for substrate recognition (Figure 4B). This suggests that the structural features of the β8–β9 hairpin may contribute to the different substrate specificity of SMYD proteins. Additionally, the *C*-terminal domain of SMYD2 is directly involved in ERα and p53 binding, but the different CTD conformation in SMYD1 and SMYD3 implies a substantially different substrate-binding mode in order to adapt to different substrate binding pockets (Figure 4C). The CTD conformation in SMYD proteins correlates with the size of the substrate-binding pocket, which may thereby provide another level of substrate specificity. Interestingly, SMYD3 appears to prefer smaller sized substrates and its methyltransferase activity is significantly higher for H4K5 and MAP3K2 than H4K5 [5,44]. This is likely due to the small glycine residues neighboring the target lysine in H4K5 and MAP3K2. The smallest and most flexible amino acid, glycine, may facilitate substrate access to the closed active site of SMYD3 (Figure 4C).

Comparison of the SMYD2–ERα and SMYD2–p53 structures has provided insight into the broad substrate specificity of SMYD2 [32]. SMYD2 is able to methylate several targets and the structural basis of this broad substrate specificity lies in the presence of multiple substrate-binding sites in SMYD2 structure [29,32,33]. The structural comparison shows that the liganded SMYD2 structures are well superimposed with an RMSD value of 0.6 Å out of 430 C_α_ atoms [32]. The ERα and p53 peptides have a similar U-shape conformation and are well superimposed at positions −1, 0, +1, and +2 (position 0 referring to the target lysine) (Figure 4D). Large deviations are found in the peptides extending out of the U-base. For example, different interactions are seen at positions +3 and +5 between the ERα and p53 peptides (Figure, 4D). Arg+3 and Arg+5 in the ERα peptide binds to the β8–β9 region in the SET domain. In the p53 peptide, Lys+3 interacts with Tyr370, Tyr374, and Asp242, and the Gln+5 chain is inserted into a pocket formed by His341, Tyr344, Gln345, Tyr370, Leu244, and Tyr245. The two different peptide conformations and the broad substrate binding space inside SMYD2 present plausible explanations for the multiple accommodations for SMYD2 and substrate binding. Structural study of additional SMYD2–substrate complexes may be necessary to corroborate this model. Because of the sequence diversity of SMYD2 substrates [32], the new SMYD2–substrate complex structures could potentially lead to the identification of novel substrate binding modes.

### 2.6. Target Lysine Access Channel

**Figure 5:**
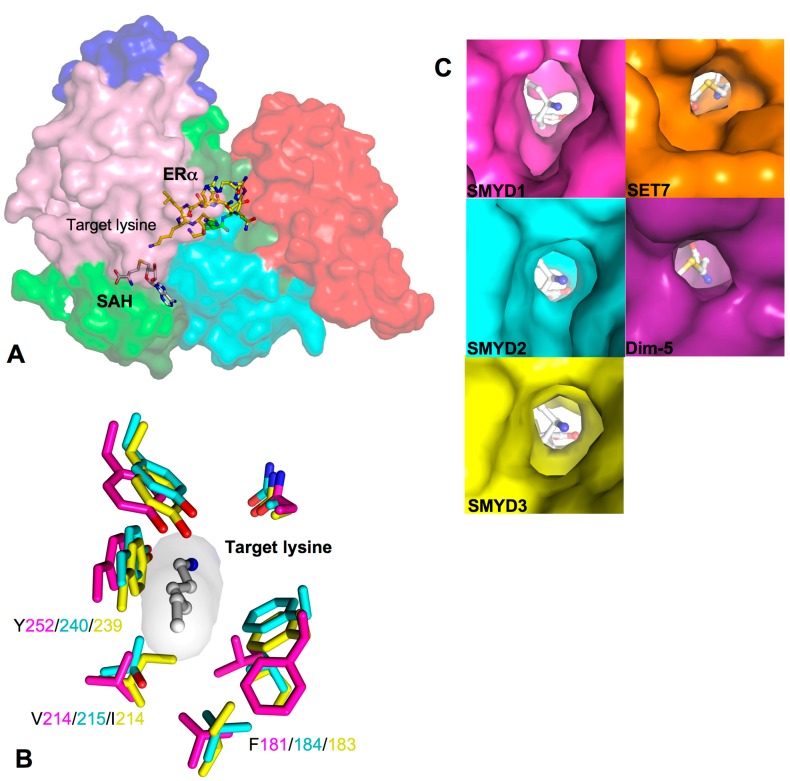
Target lysine access channel. (**A**) Surface representation of overall SMYD2–ERα structure. ERα peptide, SAH, and target lysine are indicated; (**B**) Superposition of the lysine access channels. SMYD residues are represented by sticks with the carbon atoms colored according to the scheme in Figure 1C. Target lysine is colored in white; and (**C**) Surface representation of the lysine access channel of SMYD1–3, SET7, and Dim-5. SAH or SFG is depicted by sticks with the carbon atoms colored in white.

In SET domain-containing enzymes, the lysine targeted for methylation fits into a hydrophobic pocket called the target lysine access channel (Figure 5A). The lysine access channel in SMYD proteins has a well-superimposed backbone and consists of a hydrophobic core surrounding the hydrophobic portion of the targeted lysine (Figure 5B). A highly conserved tyrosine residue preceding the post-SET domain is found in all SET classes including SMYD1 (Tyr252), SMYD2 (Tyr240), and SMYD3 (Tyr239). The tyrosine side chain appears to orient the target lysine into the channel, and substitution of this residue to phenylalanine completely abolished the enzymatic activity of SMYD2 and SMYD3 [3,30,47]. Seven residues are important for creating the lysine crevice, and of the seven, only three are aromatic residues. Two tyrosines and one phenylalanine are well conserved among SMYD1–3 with the exception of the orientation of phenylalanine in SMYD1. The aromatic ring is rotated about 110° around the C_α_–C_β_ bond axis which is pointed away from the lysine access channel; therefore, SMYD2 and SMYD3 have a well-defined and tighter channel than SMYD1 (Figure 5C). The other three small and non-aromatic residues are fairly conserved and responsible for creating a more open channel in comparison to SET7 and Dim-5 [7].

A more open channel may accommodate a larger substrate or is susceptible to ligand-induced conformational changes. It may also contribute to the broad and weak substrate binding especially for H3K4 methylation. This weak binding appears to be largely contributed to the substitution of large aromatic residues to smaller hydrophobic side chains such as valine, leucine, and isoleucine [7]. For example, substitution of Val214 to tyrosine in SMYD1 created a tighter access pocket, and thereby the binding of SMYD1 and H3 peptide significantly increased presumably due to a more compact channel and the additional hydrogen bond between the tyrosine hydroxyl group and an ε-amino from the neighboring lysine side chain [7]. This gain-of-function mutation indicates that a well-defined channel is important for securing the target lysine in the active site. A ligand-induced conformational change in the lysine access channel may be required for SMYD1 to efficiently methylate a substrate. In order to address these questions, it is of interest to determine a SMYD1 structure in complex with a substrate.

### 2.7. TPR-Like C-Terminal Domain

**Figure 6:**
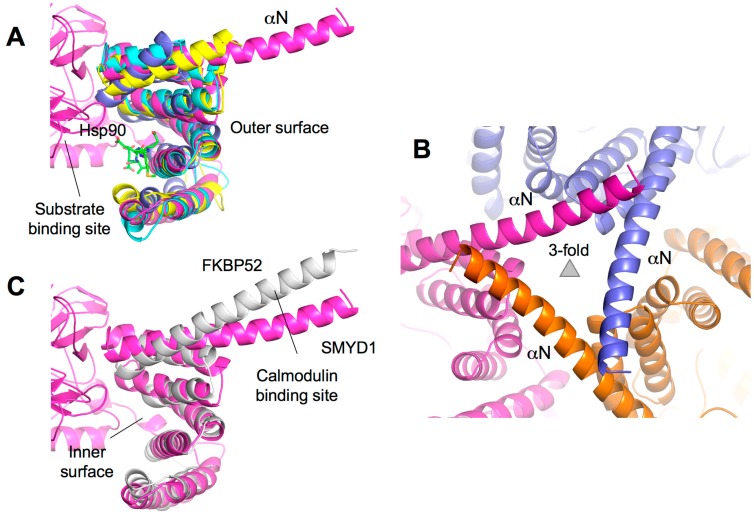
TPR-like *C*-terminal domain (CTD). (**A**) Structural superposition of the CTD domains of SMYD and TPR domain of Hop1 (PDB code: 1ELR). SMYD proteins are represented by ribbons and colored according to the scheme in Figure 1C. The Hop1 TPR domain is shown in blue. Hsp90 peptide bound to Hop1 is depicted by balls-and-sticks; (**B**) Crystal lattice of SMYD1 shows the involvement of the protruding *C*-terminal α-helix in the crystal packing; and (**C**) Structural superposition of the CTD domain of SMYD1 and TPR domain of FKBP52 (PDB code: 1QZ2). SMYD1 is colored in purple and FKBP52 in white.

The CTD domain of SMYD proteins contains a series of antiparallel α-helices that display a similar structure to TPR domains despite the lack of sequence identity (Figure 1B). TPR domains are important for binding of cochaperones to Hsp90. For example, the Hop1 TPR domain binds to the very *C*-terminal end of Hsp90 mediating Hsp90 chaperone activity (Figure 6A) [48,49]. The CTD structure is also well conserved in the SMYD family, and the only chief difference is the extended and protruded αN helix that resembles the “handle” of the wrench-shaped SMYD1 (Figure 6A). This feature is unique to SMYD1, as SMYD2–3 with the shorter αN helix resemble the shape of a clam-like shell. The unique portion of the αN helix in SMYD1 is well conserved from fish to human (data not shown). This region contains a patch of hydrophobic residues that mediate the crystal packing in SMYD1 crystals (Figure 6B). Interestingly, the CTD structure of SMYD1 is similar to the TPR structure of FKBP52 (Figure 6C). In FKBP52, the TPR domain also has a protruding *C*-terminal helix that contains a putative binding site for calmodulin [50,51]. The function of the unique SMYD1 *C*-terminal helical tail is unknown, but the conserved sequence and involvement in the crystal packing suggests that it may serve as a site for protein–protein interaction.

The structural orientation of the CTD varies significantly among the SMYD family (Figure 1C). SMYD1 has an open CTD conformation with the substrate-binding cleft completely exposed in the protein. In SMYD3, the CTD conformation is closed as the significant contact between the *N*- and *C*-terminal lobes creates a narrower opening to the substrate-binding pocket [11]. SMYD2 is like a conformational intermediate between SMYD1 and SMYD3. Additionally, the CTD domain of SMYD2 is flexible and can undergo a conformational change when different cofactors bind [12]. The CTD conformational change results in two SMYD2 structures with a slight difference in the size and shape of the substrate-binding pocket [12]. Therefore, the orientation of the CTD domain may affect substrate specificity, and different pocket shapes and sizes may be involved in modulating the substrate preference of SMYD proteins. In addition, the CTD flexibility of SMYD2 suggests that SMYD2 may have the ability to adapt to substrates with different sizes, implying broad substrate specificity. Interestingly, SMYD2 so far has the broadest substrate specificity among SMYD proteins [32].

The CTD domain appears to play dual roles in substrate binding. Deletion of the CTD from SMYD1 results in increased binding and methylation on histone H3 suggesting that the CTD may have steric effects controlling substrate access to the active site [7]. In SMYD2, the CTD plays an important role in substrate recognition and binding, but the actual effect of the CTD appears to be substrate-dependent. The CTD deletion has no effect on methylation of p53 peptide and histone H3 protein, but it results in a significant increase in H3K4 peptide methylation and significant decrease in p53 protein methylation [33]. Although these results seem paradoxical, the differential CTD effects have proved its complex roles in substrate recognition and binding. In addition, the TPR-like structure of the CTD suggests a potential role for the CTD in modulating protein–protein interaction. The predicted peptide-binding site in the CTD is located at the inner surface of the CTD in close proximity to the substrate-binding pocket (Figure 6A) [12,32]. Binding to this location could therefore affect the substrate binding and enzymatic activity. Interestingly, the activity of SMYD proteins can be significantly increased in the presence of Hsp90 [2,14,15]. In the case of SMYD2, Hsp90 not only enhances the activity but also changes the substrate preference from H3K36 to H3K4 [14]. The questions remain whether Hsp90 regulates SMYD function via binding to the CTD domain and whether such binding has a reciprocal effect on Hsp90 chaperone activity. Nonetheless, the close proximity to the active site and flexibility and multi-orientations of the CTD suggest that it is a bona fide regulatory motif in SMYD proteins.

### 2.8. Additional Substrate Binding Site?

**Figure 7:**
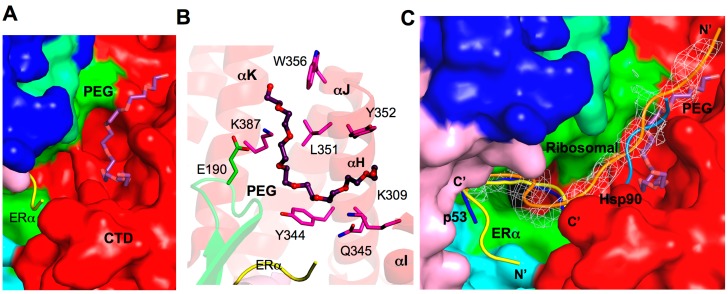
Additional substrate binding site. (**A**) Surface representation of the PEG binding site in SMYD2. PEG is depicted by sticks with the carbon atoms colored in purple. ERα peptide is displayed as ribbon and colored yellow; (**B**) Putative PEG interacting residues. SMYD2 residues are colored according to domains. ERα residues are shown in yellow. PEG is represented in the same way as in (**A**); and (**C**) Comparison of the binding sites of ERα (yellow), p53 (blue), PEG (purple), Hsp90 (light blue), and a ribosomal peptide (orange). The ribosomal peptide is overlaid with 2*F_o_*−*F_c_* omit map calculated at 2.8 Å and contoured at 1.5σ. The Hsp90 peptide is modeled by superposition of the SMYD2 CTD and Hop1 TPR.

The polyethylene glycol (PEG)-binding site found in the SMYD2–ERα structure has suggested additional and extended substrate-binding pockets (Figure 7A) [32]. PEG binding was also found in other protein structures, and in most cases, PEG binding has important functional implications mimicking ligand binding in proteins [52,53,54]. In SMYD2, the PEG molecule primarily binds to the CTD domain with an omega-turn conformation with one end found near the surface groove shaped by αH, αI, and αJ and the other end extended between αK and αL helices (Figure 7B). The residues responsible for contributing PEG binding include Lys309, Tyr344, Gln345, Gly348, Leu351, Tyr352, Trp356, and Lys387 from the CTD and Glu190 from the SET domain. The ERα peptide may also interact and stabilize PEG binding due to its close proximity to Arg+3. Note that all of the residues participating in PEG binding (except for Lys309) are not conserved in the SMYD family, which indicates a possible SMYD2-specific binding site.

The PEG binding site overlaps the predicted Hsp90 binding site (Figure 7C), suggesting that the PEG binding site might possess the peptide or substrate binding potential. This notion is supported by the SMYD2 structure binding to a ribosomal peptide (unpublished data). Instead of forming a U-shape formed by the ERα and p53 peptides, the ribosomal peptide density creates a partial U-shape at the ERα or p53-binding pocket, but the *N*-terminal end extends out through the αH/αI/αJ-binding groove of the PEG molecule (Figure 7C). This new mode of binding is vastly different to the ERα and p53 peptides and the orientations of the peptides are completely reversed. This demonstrates the exceptional substrate adaptability of SMYD2, and the multiple binding sites and that some of these binding sites may be substrate-specific have provided explanations for its broad substrate specificity. It is of interest to reveal whether the αK/αL-binding groove of the PEG molecule in SMYD2 also indicate an additional substrate-binding pocket. Further investigation into different peptide binding and conformations are necessary to better characterize the diversity of SMYD structure and function.

## 3. Drug Design Perspective

SMYD proteins provide a new avenue for cancer and cardiovascular treatment. Overexpression of several of SMYD proteins is associated with nearly all cancer types [5,24,25,41,55]. Overexpression of SMYD1 represses transcription of genes necessary to produce ion channels in the heart, and repression of ion channel expression causes heart failure [56]. SMYD1 overexpression was also found in hypoplastic left heart syndrome (HLHS), a disorder characterized by the severely underdeveloped left ventricle [57]. SMYD2 is overexpressed in ESCC or p53-related cancers, and knockdown of SMYD2 inhibits tumor cell proliferation [24,25,55,58]. SMYD3 is overexpressed in more than 14 types of cancers such as breast cancer, colon cancer, prostate cancer, lung cancer, and pancreatic cancer [2,41,59,60,61,62,63,64,65,66,67,68]. SMYD3 overexpression often correlates with poor prognosis, and knockdown of SMYD3 proved to inhibit tumor growth [2,64,65,66,67,68,69]. Therefore, drug intervention of any of SMYD proteins may be beneficial to the fields of cardiovascular disease and cancer.

**Figure 8:**
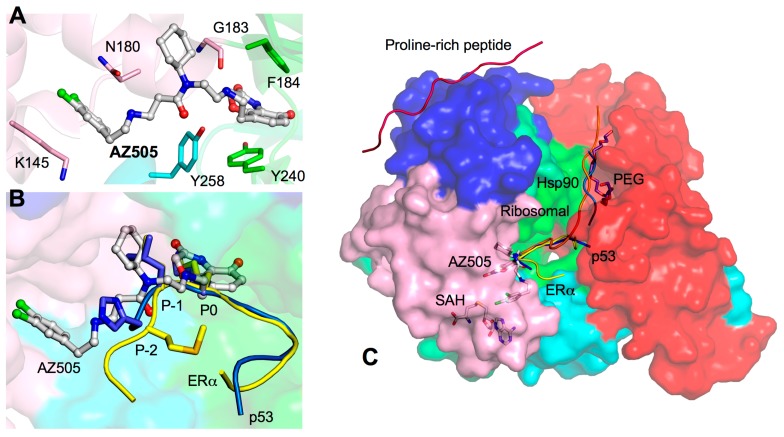
AZ505-bound SMYD2 structure. (**A**) Interactions between AZ505 and SMYD2. AZ505 is depicted by balls-and-sticks with the carbon atoms colored in white. SMYD2 residues are depicted by sticks colored according to domains; (**B**) Comparison of the binding sites of ERα, p53, and AZ505. The ERα and p53 peptides are depicted by sticks and colored in yellow and blue. AZ505 is represented in the same way as in (**A**); and (**C**) Surface representation of SMYD2–ERα structure illustrates potential drug targeting sites: ERα (yellow), p53 (blue), PEG (purple), Hsp90 (light blue), the ribosomal peptide (orange), and proline-rich peptide (hot pink). SAH and AZ505 are depicted by sticks.

Efforts to create SMYD inhibitors are currently underway. AZ505 is a potent SMYD2 competitive inhibitor recently identified from a high throughput chemical screening [29]. In the crystal structure, AZ505 bound to the lysine access channel, and ITC analysis indicated inhibitor binding is primarily driven by hydrophobic interactions providing a low K_D_ ~0.5 μM (Figure 8A) [29]. The three moieties of AZ505 have similar interactions found in the p53 and ERα peptides. The benzooxazinone group is packaged into the lysine channel where several hydrophobic and electrostatic interactions are made (Figure 8A). The cyclohexyl and dichlorophenethyl groups adopt the same −1 and −2 position, but they appear more compact to the surface than the p53 and ERα peptides (Figure 8B). In addition, the Gly183 carbonyl oxygen forms a similar hydrogen bond to the amide linker between the benzooxazinone and cyclohexyl groups. Therefore, the potency of AZ505 appears to be due to a complete blockage of the core region of the SMYD2 active site and preventing it from binding to the target lysine.

Therapeutic drug intervention of SMYD proteins may not be limited to inhibiting the lysine access channel. SMYD2 is necessary for methylating many targets and is involved in various functionally independent cellular processes [32]. Complete knockdown of SMYD2 may not be a viable option since unselective SMYD2 inhibition may cause undesirable and perhaps lethal side effects. In order to selectively design a therapeutic drug to inhibit SMYD2 function in the context of cancer, one may consider targeting alternative binding sites for inhibition that will interfere with only a subset population of SMYD2. For example, designing a drug that will mimic the binding properties of the p53 peptide may provide specificity to oncogenic SMYD2 function in p53-related cancers. Targeting the binding properties of the ERα peptide may be beneficial to aggressive ERα-negative breast tumors by specifically restoring functional ERα expression [47]. The PEG or ribosomal binding site may also provide a genuine substrate-specific targeting option without the fear of interference with binding of the p53 and ERα peptides. Targeting MYND-mediated protein interactions may be another viable approach in cancer therapy as binding of the MYND to the proline-rich sequences of the tumor suppressor EBP41L3 links SMYD2 to meningiomas and lung cancer [14,70,71]. Finally, the CTD orientations related to the sizes and shapes of the SMYD substrate-binding pockets raise a possibility for selective drug design for different members of the SMYD family. Challenges remain because of the potential conformational flexibility of the CTD domain of SMYD proteins. Selective and potent drug design will require the consideration of conformational changes in SMYD proteins and understanding of the functional role of each conformational state. Further investigation into the structural differences between SMYD proteins will be necessary to distinguish specificity and efficacy into the drug design process.

## 4. Concluding Remarks

SMYD proteins are an exciting field of study as they are linked to many types of cancer-related pathways. Cardiac and skeletal muscle development and function also depend on SMYD proteins opening a possible avenue for cardiac-related treatment. The purpose of this review is to gather current structural data to support the versatile roles of SMYD proteins. We provide a summary of the structures of the SMYD family focusing on their structural differences. The structures of the individual domains in SMYD1–3 are similar but the orientations of the CTD are substantially different resulting in open or closed conformations. Different CTD conformations suggest that SMYD proteins could undergo a conformational change that offers dynamics for regulation of substrate specificity [7,11,12]. SMYD2 conformations are sensitive to cofactor binding which alters the size of the substrate-binding pocket [12]. It is conceivable that the methyltransferase activity of SMYD proteins may be regulated by controlling lobe conformation and dynamics like some kinases [72,73]. SMYD structures have a potential for efficacious drug intervention, but efforts to design a drug should not be limited to the target lysine access channel. Analysis of SMYD structures revealed many other binding sites with drug targeting potential, such as the broad substrate-binding pocket, PEG and ribosomal binding site, proline-rich peptide binding site, and a yet-unidentified DNA binding groove (Figure 8C). With the different binding sites and conformations, it is possible to effectively knockdown cancerous function of SMYD proteins such as SMYD2 and SMYD3 without disrupting the entire functional population of SMYD proteins. Additionally, analysis of current structures raised many new questions. The unique protruding *C*-terminal helix of SMYD1 may be involved in protein–protein interaction. The CTD orientation may determine substrate specificity. How DNA binding alters SMYD3 structure and function remains to be determined. Whether the possible binding of Hsp90 to the CTD provides a mechanism for SMYD activity enhancement and the potential role of this coplay in cancer and heart development are also unclear. In summary, SMYD proteins are of functional and therapeutic importance, and continued elucidation of their structural differences and substrate specificity will lead to additional functional implications.
